# Hemoglobin-to-Red Cell Distribution Width Ratio Independently Predicts Survival in Non-Metastatic Gastric Cancer: A Retrospective Cohort Study

**DOI:** 10.3390/medicina62091797

**Published:** 2026-09-18

**Authors:** Sevgi Bilmez Altay, İlker Nihat Ökten, Çağlar Köseoğlu, Ertürk Yiğit, Mehmet Uzun, Oğuzhan Kesen

**Affiliations:** 1 Department of Internal Medicine, Gaziantep Özel Emek Hospital, Gaziantep 27060, Turkey; 2 Department of Medical Oncology, Göztepe Prof. Dr. Süleyman Yalçın City Hospital, Istanbul 34722, Turkey; ilkernihat@gmail.com; 3 Department of Medical Oncology, Gaziantep City Hospital, Gaziantep 27470, Turkey; drcaglarkoseoglu@hotmail.com (Ç.K.); drerturkyigit@hotmail.com (E.Y.); 4 Department of Medical Oncology, University of Health Sciences, İzmir Tepecik Training and Research Hospital, Izmir 35020, Turkey; memed.uzun3846@gmail.com; 5 Department of Medical Oncology, Recep Tayyip Erdoğan University, Rize 53100, Turkey; oguzhan.kesen@erdogan.edu.tr

**Keywords:** hemoglobin/red cell distribution width (HGB/RDW) ratio, gastric cancer, overall survival, progression-free survival, prognostic biomarker

## Abstract

*Background and Objectives*: Gastric cancer is one of the most common malignancies worldwide and continues to be associated with high mortality. The hemoglobin-to-red cell distribution width (HGB/RDW) ratio has emerged as a promising hematological prognostic biomarker in various cancers. However, its prognostic significance in patients with curative-intent resection of non-metastatic gastric cancer remains insufficiently investigated. This study aimed to evaluate the association between the pretreatment HGB/RDW ratio and progression-free survival (PFS) as well as overall survival (OS) in patients with non-metastatic gastric cancer. *Materials and Methods*: In this multicenter study, a total of 175 patients with non-metastatic gastric cancer who received perioperative FLOT chemotherapy (four preoperative and four postoperative cycles) and underwent curative-intent surgical resection were retrospectively analyzed. A cut-off value for the HGB/RDW ratio was calculated using receiver operating characteristic (ROC) curve analysis. Survival outcomes, including progression-free survival (PFS) and overall survival (OS), were analyzed using the Kaplan–Meier method. Multivariate Cox proportional hazards regression analysis was performed to identify independent prognostic factors. *Results*: Patients with an HGB/RDW ratio > 0.85 demonstrated significantly superior 2-year and 5-year OS rates (76.2% vs. 59.6% and 51.6% vs. 27.7%, respectively; *p* = 0.003) and PFS rates (69.4% vs. 49.6% and 53.7% vs. 36.4%, respectively; *p* = 0.047) compared to those with HGB/RDW ≤ 0.85. Multivariate analysis identified HGB/RDW ≤ 0.85 as an independent predictor of both increased mortality risk (HR: 1.88, 95% CI: 1.16–3.06; *p* = 0.011) and disease progression risk (HR: 1.79, 95% CI: 1.18–2.72; *p* = 0.006). *Conclusions*: The pretreatment HGB/RDW ratio is an independent prognostic factor for OS and PFS in patients with curative-intent resection of non-metastatic gastric cancer and may be considered a practical marker that could contribute to treatment planning in this patient population.

## 1. Introduction

Gastric cancer remains a major global health burden, ranking sixth in incidence among all cancers and accounting for 5.6% of newly diagnosed cases according to GLOBOCAN 2020 data; it also represents the third leading cause of cancer-related mortality, responsible for 7.7% of all cancer deaths [1]. Owing to the absence of specific clinical manifestations in early-stage gastric cancer, many patients are diagnosed at an advanced stage, and more than 60% present with advanced disease at initial diagnosis [2]. Despite advances in systemic chemotherapy and targeted therapies, the prognosis of patients with advanced gastric cancer remains poor, with a 5-year survival rate of approximately 10% for those who are not candidates for surgical treatment [1,2]. Therefore, the identification of easily accessible and reliable prognostic biomarkers remains an important clinical need. In this context, prognostic indicators derived from routine hematological parameters are gaining increasing importance in oncology. Biomarkers obtained from standard complete blood count (CBC) tests—including hemoglobin (HGB), red cell distribution width (RDW), neutrophil-to-lymphocyte ratio (NLR), platelet-to-lymphocyte ratio (PLR), and the systemic immune–inflammation index (SII)—have been widely investigated as potential indicators of disease prognosis [3].

Hemoglobin is a critical physiological parameter, and its decrease in patients with cancer is often multifactorial, reflecting mechanisms such as chronic inflammation, nutritional deficiency, bone marrow suppression, and tumor-associated bleeding. Reduced HGB levels contribute to tumor hypoxia, which in turn promotes angiogenesis and facilitates the acquisition of a more aggressive tumor phenotype and poor clinical outcomes [4,5,6,7]. RDW, another parameter derived from routine hemogram analysis, reflects the variability in red blood cell volume. Increasing evidence suggests that elevated RDW levels are associated with systemic inflammation, impaired erythropoiesis, and oxidative stress—all of which may promote tumorigenesis and disease progression [8]. Accordingly, RDW has been shown to serve as both a diagnostic and prognostic biomarker in hematological malignancies and solid tumors [8].

However, HGB and RDW levels individually may be influenced by various clinical and biological factors unrelated to malignancy. To mitigate this limitation, the hemoglobin-to-red cell distribution width ratio (HGB/RDW) has been proposed as a composite biomarker with enhanced specificity and sensitivity compared to each parameter alone [9,10,11]. The HGB/RDW ratio may serve as an easily accessible composite index that reflects nutritional status, immune function, and inflammatory status. Recent studies have demonstrated that a low HGB/RDW ratio is associated with poor prognosis in many malignancies, including lung cancer [9,10], esophageal cancer [11], hepatocellular carcinoma [12], colorectal cancer [13], renal cell carcinoma [14], urothelial carcinoma [15], and nasopharyngeal cancer [16]. Furthermore, a systematic review and meta-analysis demonstrated that a low HGB/RDW ratio was associated with a 2-fold increased risk of malignant events such as recurrence and metastasis [8].

Despite growing evidence across tumor types, the prognostic role of HGB/RDW in gastric cancer remains insufficiently investigated, with existing evidence derived predominantly from heterogeneous or metastatic patient populations. This limitation of previous studies is particularly relevant for patients with non-metastatic disease, who may benefit most from preoperative risk classification to optimize postoperative and adjuvant treatment strategies. To the best of our knowledge, the present study is among the first to specifically evaluate the prognostic significance of the HGB/RDW ratio in a well-defined cohort of patients with curative-intent resection of non-metastatic gastric cancer.

This study aimed to evaluate the association between the pretreatment HGB/RDW ratio and progression-free survival (PFS) as well as overall survival (OS) in patients with non-metastatic gastric cancer.

## 2. Materials and Methods

### 2.1. Patient Selection and Study Design

In this multicenter retrospective study, patients diagnosed with gastric cancer and treated at the oncology clinics of participating centers between April 2018 and April 2026 were identified using institutional hospital data systems. The study was conducted in accordance with the Declaration of Helsinki, and ethical approval was obtained from the local ethics committee (Approval No.: 538/2026). Because of the retrospective observational design, the ethics committee waived the requirement for written informed consent.

All patients received perioperative FLOT chemotherapy (fluorouracil, leucovorin, oxaliplatin, and docetaxel), consisting of four preoperative cycles administered every two weeks, followed by curative-intent gastrectomy and four additional postoperative cycles. Pathological staging was therefore reported using the post-neoadjuvant (yp) classification.

### 2.2. Inclusion and Exclusion Criteria

Surgical procedures included subtotal or total gastrectomy with D1 or D2 lymph node dissection, and resection margins were classified as R0 or R1.

Inclusion criteria were (1) age ≥ 18 years; (2) pathologically confirmed gastric adenocarcinoma; (3) non-metastatic disease (M0) at diagnosis according to the AJCC 8th edition, established by contrast-enhanced thoraco-abdominal CT, with PET/CT and/or staging laparoscopy where clinically indicated; (4) completion of perioperative FLOT chemotherapy and curative-intent gastrectomy with R0 or R1 resection; (5) a complete blood count obtained within 4 weeks before the first cycle of chemotherapy and before any transfusion or treatment for anemia; and (6) available follow-up data.

Exclusion criteria were (1) a second primary malignancy; (2) prior hematological malignancy; (3) red cell transfusion within 4 weeks before blood sampling; and (4) R2 resection or distant metastasis identified intraoperatively or on postoperative staging.

These criteria were chosen to define a population that is as homogeneous as possible in terms of treatment, staging, and the timing of blood sampling. Restricting the cohort to patients who completed perioperative FLOT and underwent curative-intent gastrectomy removes variation in chemotherapy regimen, which is the main treatment-related determinant of outcome in this setting, and means that all patients were staged with the same post-neoadjuvant (yp) system. Excluding R2 resections and intraoperatively detected metastases keeps the cohort limited to resections that were of curative intent. Selecting patients with M0 adenocarcinoma avoids the inclusion of patients in whom anemia mainly reflects metastatic burden, thus limiting the factors that can affect the HGB/RDW ratio. Requiring a complete blood count before the first cycle and before any transfusion or treatment for anemia means the ratio was measured at the same point in the treatment pathway in every patient, and a recent transfusion was excluded for the same reason, since it raises hemoglobin and widens RDW at once. A second primary malignancy was excluded as a competing cause of death, and prior hematological malignancy was excluded because it directly alters both components of the ratio.

No a priori sample size calculation was performed because this retrospective cohort study included all consecutive eligible patients treated between April 2018 and April 2026. The cohort therefore represents the complete eligible population over a fixed study period rather than a selected sample.

### 2.3. Data Collection and Definitions

Data were collected retrospectively from the hospital data systems of the participating centers by the authors of the study. Electronic medical records, laboratory data, radiology and pathology reports, and follow-up notes from patients’ oncology records were reviewed. Relevant variables were recorded using a predefined, standardized data-collection form. The HGB/RDW ratio was calculated using HGB and RDW values obtained from baseline blood tests before treatment. The HGB/RDW ratio was calculated as hemoglobin in g/dL divided by RDW-CV expressed as a percentage. The optimal cut-off value was determined by ROC curve analysis. Tumor staging was performed according to the 8th edition of the AJCC TNM staging system. Radiological treatment response was evaluated according to the Response Evaluation Criteria in Solid Tumors (RECIST), version 1.1. Histological subtype was classified according to the WHO Classification of Digestive System Tumors (5th edition), and tumors were additionally classified by the Lauren system as intestinal, diffuse, or indeterminate.

Survival data were obtained from outpatient follow-up visits and inpatient medical records until the final data collection date. PFS was defined as the time from treatment initiation to disease progression or death from any cause. OS was defined as the time from the date of diagnosis to the last follow-up visit or death from any cause, expressed in months.

### 2.4. Statistical Analysis

Statistical analyses were performed using IBM SPSS Statistics for Windows, Version 25.0 (IBM Corp., Armonk, NY, USA). Descriptive statistics are presented as numbers and percentages (%) for categorical variables and as mean ± standard deviation (SD) for continuous variables. Variables with *p* < 0.20 in univariate analysis were included in the multivariate Cox regression model. In the revised analysis, established clinicopathological factors (ypN category, histological type, and Lauren’s classification) were additionally included in the final multivariable models. Anemia was defined using sex-specific World Health Organization thresholds (hemoglobin < 13 g/dL in men and <12 g/dL in women), and the incremental prognostic value of the HGB/RDW ratio beyond anemia was evaluated by entering both variables into the Cox models. The median follow-up duration was estimated using the reverse Kaplan–Meier method. ROC curve analysis was performed to evaluate the predictive value of various indices for mortality. The optimal cut-off value for the HGB/RDW ratio was determined by ROC curve analysis using overall mortality as the state variable and was selected as the value maximizing the Youden index. Survival outcomes were compared between clinical groups using the Kaplan–Meier method with the log-rank test. Multivariate Cox proportional hazards regression analysis was used to evaluate independent predictors of mortality and disease progression. A *p* value < 0.05 was considered statistically significant.

## 3. Results

A total of 175 patients were included in the study. All patients underwent curative-intent surgical resection, including subtotal (*n* = 78) or total gastrectomy (*n* = 97), with D1 (*n* = 87) or D2 (*n* = 88) lymph node dissection. Resection margins were pathologically confirmed as R0 (*n* = 167) or R1 (*n* = 8). No patient had macroscopically positive margins (R2 resection) or distant metastasis at the time of surgery. The demographic and clinicopathological characteristics of patients according to the HGB/RDW ratio are summarized in Table 1. A significant difference was observed in the sex distribution between the groups (*p* = 0.014), with a greater proportion of female patients in the low-HGB/RDW group. Regarding histopathological subtypes, tubular/papillary adenocarcinoma not otherwise specified was more frequent in the low-HGB/RDW group, whereas poorly cohesive carcinoma of the signet-ring cell type was more frequent in the high-HGB/RDW group (*p* = 0.011). According to the Lauren classification, the intestinal type was more prevalent in the low-HGB/RDW group, while the diffuse type was more frequently observed in the high-HGB/RDW group (*p* = 0.024). A significant difference was also observed in the ypN category (*p* = 0.002), with a higher proportion of ypN2 disease in the low-HGB/RDW group. Mortality was significantly higher in the low-HGB/RDW group (*p* = 0.004). No significant differences were found between the groups with respect to clinical stage at diagnosis, tumor grade, type of surgery, response to treatment, or surgical margin status (all *p* > 0.05).

Anemia, defined by sex-specific WHO thresholds, was present in 89 patients (50.9%) and was strongly concentrated in the low-HGB/RDW group (91.6% vs. 14.1% in the high group; *p* < 0.001). When anemia and the HGB/RDW ratio were entered jointly into the Cox models, anemia alone was not independently associated with outcome (overall survival, *p* = 0.90; progression-free survival, *p* = 0.57), whereas a low HGB/RDW ratio remained an independent predictor of both overall survival (HR 1.96, 95% CI 1.01–3.80; *p* = 0.047) and progression-free survival (HR 2.12, 95% CI 1.16–3.88; *p* = 0.015). These findings indicate that the HGB/RDW ratio provides prognostic information beyond a simple binary anemia classification.

**Figure 1:**
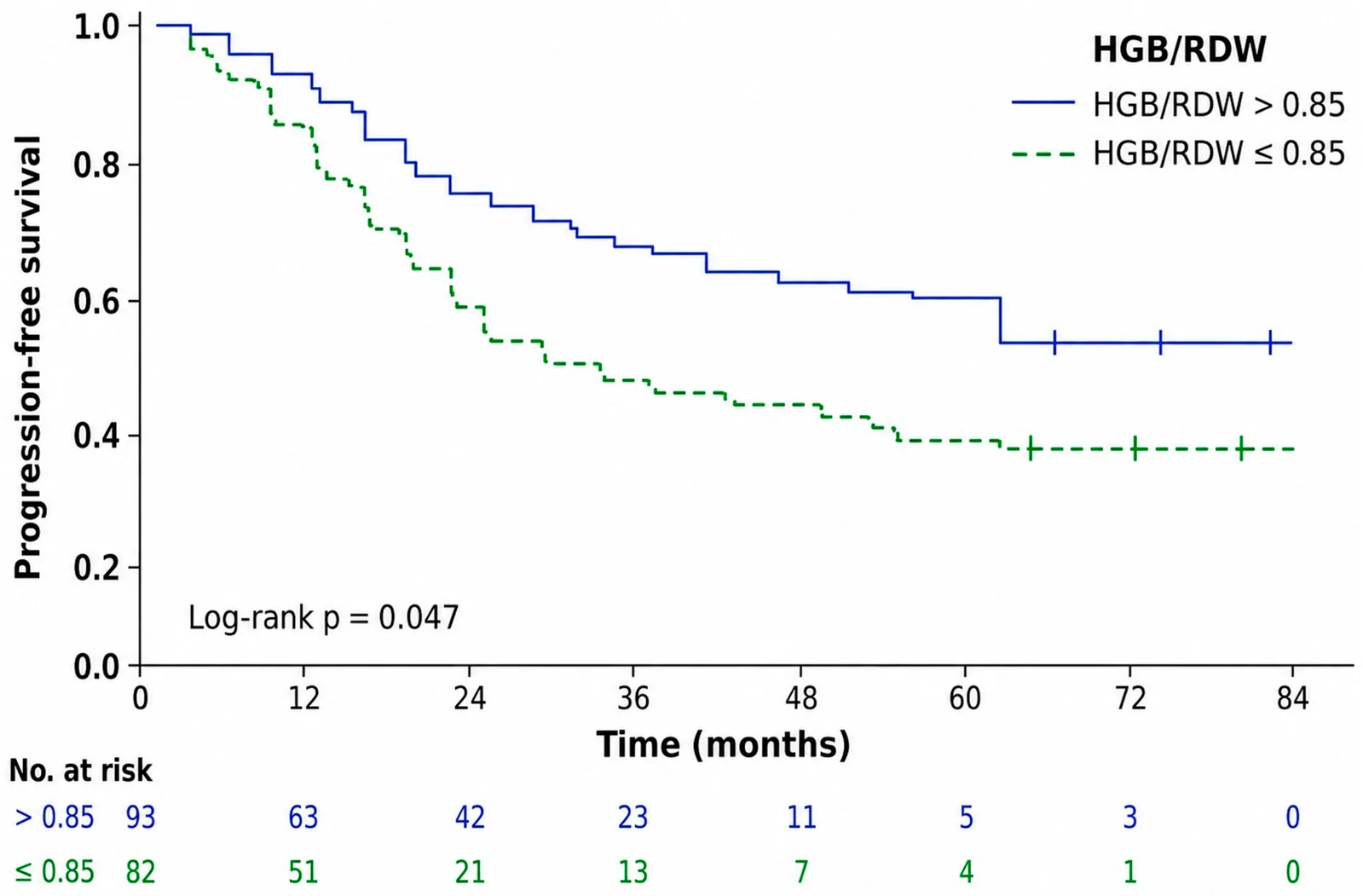
Kaplan–Meier progression-free survival curves stratified by the HGB/RDW ratio (>0.85 vs. ≤0.85).

The discriminative value of the pretreatment hematological indices for overall mortality is shown in Table 2. Baseline inflammatory and nutritional indices differed between the two HGB/RDW groups. Patients with a low HGB/RDW ratio had a higher systemic immune–inflammation index (median 865 vs. 700; *p* = 0.021), a higher C-reactive protein-to-albumin ratio (1.60 vs. 1.10; *p* = 0.006), a higher pan-immune–inflammation value (518 vs. 382; *p* = 0.020), and a higher platelet-to-albumin ratio (7.94 vs. 6.37; *p* < 0.001), together with a lower prognostic nutritional index (46.48 ± 7.51 vs. 49.64 ± 6.68; *p* = 0.004). Body mass index (24.36 vs. 24.62; *p* = 0.373), the neutrophil-to-lymphocyte ratio (2.84 vs. 2.62; *p* = 0.208), and the lymphocyte-to-monocyte ratio (3.00 vs. 3.18; *p* = 0.054) did not differ significantly between groups. The median follow-up duration was 34.1 months (interquartile range 18.3–53.6; range 3.2–83.4). The Kaplan–Meier PFS analyses are presented in Table 3. In the overall cohort, the 2-year and 5-year PFS rates were 60.7% and 42.3%, respectively, and the median PFS was 38.46 months (95% CI: 16.13–60.79). The HGB/RDW ratio demonstrated a significant association with PFS (*p* = 0.047). In ROC curve analysis for overall mortality, the HGB/RDW ratio yielded an area under the curve of 0.608 (95% CI 0.524–0.693; *p* = 0.014). The optimal cut-off value was 0.85, with a sensitivity of 60.0% and a specificity of 62.0% (Youden index 0.22). Patients with an HGB/RDW ratio > 0.85 had 2-year and 5-year PFS rates of 69.4% and 53.7%, respectively, whereas those with a ratio ≤ 0.85 had rates of 49.6% and 36.4%. The Kaplan–Meier curves showed a clear and sustained separation between the two groups throughout follow-up, with the high-HGB/RDW group maintaining consistently higher progression-free survival probabilities (Figure 1).

**Figure 2:**
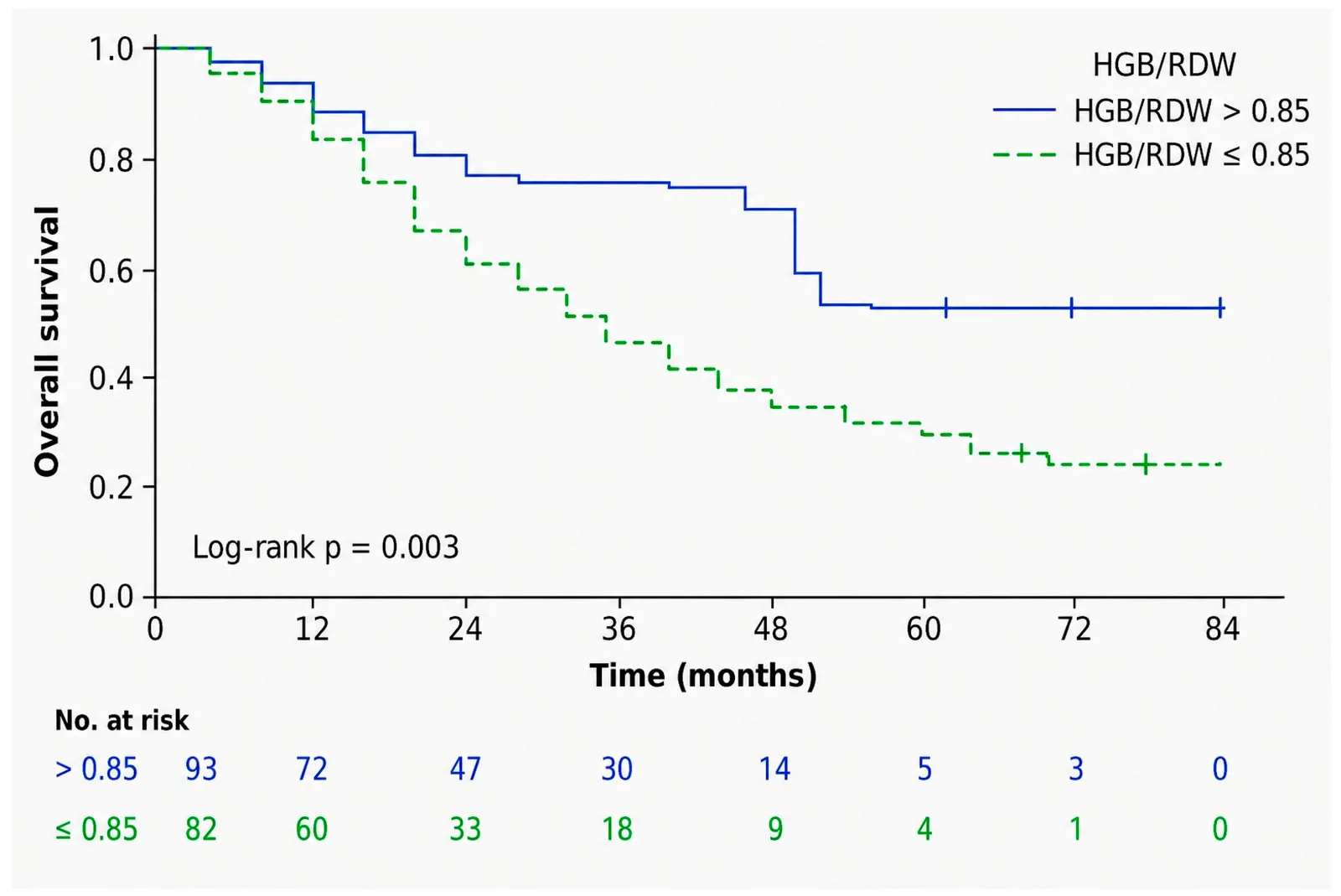
Kaplan–Meier overall survival curves stratified by the HGB/RDW ratio (>0.85 vs. ≤0.85).

The Kaplan–Meier OS analyses are summarized in Table 4. In the overall cohort, the 2-year and 5-year OS rates were 68.4% and 39.6%, respectively, and the median OS was 43.50 months (95% CI: 35.38–51.61). Patients with an HGB/RDW ratio > 0.85 had 2-year and 5-year OS rates of 76.2% and 51.6%, respectively, whereas those with a ratio ≤ 0.85 had rates of 59.6% and 27.7%. The difference between the groups was statistically significant (*p* = 0.003). The Kaplan–Meier curves demonstrated an early and persistent divergence between the groups, with patients in the high-HGB/RDW group showing a markedly higher overall survival probability over time (Figure 2).

In the multivariate Cox regression analysis for OS, sex, ypN category, histological type, and Lauren’s classification were included together with the HGB/RDW ratio in the model. HGB/RDW ≤ 0.85 was independently associated with an increased risk of mortality (HR: 1.88, 95% CI: 1.16–3.06; *p* = 0.011); a higher ypN category was also independently prognostic (*p* < 0.001), whereas histological type, Lauren’s classification, and sex were not independently significant (Table 5).

For PFS, age, sex, ypN category, histological type, and Lauren’s classification were included together with the HGB/RDW ratio in the multivariate Cox regression model. HGB/RDW ≤ 0.85 was independently associated with an increased risk of disease progression (HR: 1.79, 95% CI: 1.18–2.72; *p* = 0.006); a higher ypN category was also independently prognostic (*p* < 0.001) (Table 6).

## 4. Discussion

In this multicenter, retrospective cohort study, we investigated the prognostic value of the pretreatment HGB/RDW ratio in 175 patients with curative-intent resection of non-metastatic gastric cancer. Our results demonstrate that a low HGB/RDW ratio (≤0.85) is independently associated with significantly poor OS and PFS, even after adjustment for other relevant clinical variables. To the best of our knowledge, this is among the first studies to specifically evaluate the prognostic role of HGB/RDW in a well-defined cohort of non-metastatic gastric cancer patients treated with curative surgery and chemotherapy.

The biological rationale underlying the prognostic significance of the HGB/RDW ratio is based on the pathophysiological relationship between anemia, systemic inflammation, and tumor behavior. In cancer patients, reduced hemoglobin levels typically reflect chronic disease-related anemia, driven by inflammatory cytokine-mediated suppression of erythropoiesis, reduced erythropoietin responsiveness, and myelosuppression from the tumor or its treatment [4]. Low hemoglobin promotes tumor hypoxia, which in turn activates hypoxia-inducible factor (HIF) signaling pathways, stimulates angiogenesis via VEGF upregulation, and enhances the invasive and metastatic potential of tumor cells [5,6]. Consistent with these mechanisms, prior studies in gastric cancer patients have demonstrated that preoperative anemia is an independent risk factor for poor prognosis [7].

RDW reflects the degree of anisocytosis—heterogeneity in erythrocyte size—and is influenced by iron deficiency, vitamin B12 and folate deficiency, and dysregulated erythropoiesis driven by chronic inflammatory states [17]. Elevated RDW has been associated with increased levels of inflammatory markers, including C-reactive protein, tumor necrosis factor-alpha (TNF-α), and interleukin-6, all of which play important roles in tumor microenvironment modulation and cancer progression [17]. A large meta-analysis including nearly 20,000 cancer patients demonstrated that high RDW levels are associated with shorter OS, DFS, PFS, and recurrence-free survival across multiple tumor types, including gastric cancer [18].

Although HGB and RDW individually provide prognostic information, each may be influenced by non-malignant conditions, including autoimmune diseases, hematological disorders, and exogenous factors such as exercise and dietary habits [19]. By integrating both parameters into a single composite index, the HGB/RDW ratio may better reflect the patient’s overall inflammatory and nutritional status than either marker alone [20]. In the present cohort, the HGB/RDW ratio showed the highest discriminative value for overall mortality among the fourteen hematological indices evaluated (AUC 0.608), exceeding that of the prognostic nutritional index, the platelet-to-albumin ratio, the systemic immune–inflammation index, and the neutrophil-to-lymphocyte ratio, although discrimination was modest for all indices tested.

The prognostic value of the HGB/RDW ratio has been reported in various malignancies. In small cell lung cancer, a low baseline HGB/RDW ratio was independently associated with shorter OS and PFS in patients receiving first-line chemotherapy [9]. Similarly, in resected lung adenocarcinoma, a preoperative HGB/RDW ratio below the median cut-off was associated with a significantly higher risk of disease relapse, even after adjustment for pathological nodal involvement [10]. In esophageal squamous cell carcinoma, Sun et al. [11] first proposed the HGB/RDW ratio as a prognostic biomarker, demonstrating that a high HGB/RDW ratio was associated with superior OS in patients receiving curative treatment. In hepatocellular carcinoma, a low HGB/RDW ratio was independently predictive of poor PFS and OS and showed an inverse correlation with tumor staging, suggesting a potential mechanistic link between the ratio and tumor progression [12]. In colorectal cancer, Li et al. demonstrated that HGB/RDW was an independent predictor of both OS and PFS, with patients in the low-HGB/RDW group showing substantially worse survival outcomes [13].

The prognostic role of HGB/RDW has also been demonstrated in urological malignancies. In upper tract urothelial carcinoma, a low HGB/RDW ratio was associated with disease progression and poor prognosis [15], and similar findings were reported in metastatic renal cell carcinoma patients treated with targeted therapy [14]. In locally advanced nasopharyngeal cancer, a low HGB/RDW ratio was an independent predictor of both OS and disease-free survival (DFS) in patients receiving chemoradiotherapy, with a hazard ratio of 3.07 for OS [16].

In the context of gastric cancer, existing evidence on HGB/RDW has been predominantly derived from heterogeneous or metastatic cohorts, limiting the applicability of prior findings to patients with resectable disease. Yılmaz et al. reported that the HGB/RDW ratio predicted survival in gastric cancer patients receiving neoadjuvant FLOT chemotherapy, with a low HGB/RDW ratio associated with significantly shorter OS [21]. In addition, a meta-analysis by Chi et al. encompassing 2985 cancer patients from 11 retrospective studies confirmed that a low HGB/RDW ratio was associated with a two-fold increased risk of adverse oncological events [8].

Our findings are consistent with these reports and extend the evidence to a well-defined cohort of non-metastatic gastric cancer patients who underwent curative resection. Notably, we observed that the low-HGB/RDW group was characterized by a higher proportion of female patients, tubular/papillary histology, and intestinal-type Lauren’s classification—features that collectively suggest a distinct biological and demographic profile. The association between diffuse-type gastric cancer and a high HGB/RDW ratio in our cohort may warrant further investigation, as this histological subtype is generally associated with a more aggressive clinical course. Furthermore, a low HGB/RDW ratio was associated with a 1.88-fold increase in mortality risk and a 1.79-fold increase in progression risk in multivariate analysis, underscoring its independent prognostic value independent of nodal status (ypN category), histological type, and Lauren’s classification.

From a clinical perspective, the HGB/RDW ratio offers several practical advantages over established prognostic tools. It is derived from routine CBC parameters that are universally available, inexpensive, non-invasive, and measured as part of standard preoperative workup. Unlike complex immunohistochemical or molecular biomarkers, the HGB/RDW ratio requires no additional testing, making it particularly suitable for resource-limited settings. These characteristics align with the emerging emphasis on integrating cost-effective biomarkers into cancer staging and risk stratification frameworks.

In our cohort, women were over-represented in the low-HGB/RDW group (36.1% vs. 19.6%) yet had numerically better survival than men (2-year overall survival, 72.1% vs. 59.1%). Thus, any residual confounding by sex would be expected to weaken, rather than exaggerate, the observed association. Nevertheless, because hemoglobin levels are physiologically lower in women, sex-specific thresholds may improve the performance of hemoglobin-based indices and should be evaluated in larger cohorts.

Several limitations of the present study should be acknowledged. First, the retrospective design precluded standardization of follow-up intervals and data collection procedures. Second, the lack of a standard cut-off value across studies represents a methodological challenge, as different investigators have determined cut-off values using distinct statistical approaches and patient populations. Third, the cohort is by design a completer cohort: all patients survived preoperative chemotherapy, remained fit for surgery, and underwent curative-intent resection. Patients who progressed during neoadjuvant treatment became unfit for surgery or died before resection are therefore not represented, which limits generalizability to all patients commencing FLOT. Because a low HGB/RDW ratio is plausibly associated with the poorer performance status underlying such attrition, this selection is likely to render our estimates conservative rather than inflated. Fourth, information on iron, vitamin B12 and folate status, renal and hepatic function, recent infection, and any hematinic or erythropoiesis-stimulating treatment given before baseline sampling was not systematically available in the patients’ records. All patients had a histologically confirmed gastric adenocarcinoma requiring perioperative chemotherapy, a setting in which anemia and anisocytosis are predominantly disease- or treatment-related; nevertheless, prospective studies recording these parameters would allow the contribution of non-malignant causes of a raised RDW to be quantified. Future large-scale, multicenter prospective studies should address these limitations, standardize the HGB/RDW cut-off value, and explore the ratio’s utility in combination with other established biomarkers to improve prognostic accuracy in gastric cancer.

## 5. Conclusions

The pretreatment HGB/RDW ratio is an independent prognostic factor for OS and PFS in patients with curative-intent resection of non-metastatic gastric cancer. A low HGB/RDW ratio (≤0.85) was associated with significantly increased risks of disease progression and mortality. Given its simplicity and availability, the HGB/RDW ratio may serve as a practical marker for preoperative risk assessment.

## Figures and Tables

**Table 1:** Clinical and clinicopathological characteristics of patients according to HGB/RDW groups.

	Total	High HGB/RDW	Low HGB/RDW	*p*
Variables	*N* = 175	>0.85 (*N* = 92)	≤0.85 (*N* = 83)	
**Age**				0.058 ^a^
Mean ± SD	63.25 ± 10.33	61.80 ± 10.40	64.85 ± 10.07	
Median (min–max)	64.0 (28–85)	63.0 (28–84)	67.0 (38–85)	
**Sex**				0.014 ^b^
Female	48 (27.4)	18 (19.6)	30 (36.1)	
Male	127 (72.6)	74 (80.4)	53 (63.9)	
**Stage at diagnosis**				0.510 ^c^
I	2 (1.1)	2 (2.2)	0 (0)	
IIA	32 (18.3)	15 (16.3)	17 (20.5)	
IIB	29 (16.6)	14 (15.2)	15 (18.1)	
III	111 (63.4)	61 (66.3)	50 (60.2)	
IVA	1 (0.6)	0 (0)	1 (1.2)	
**Histological type**				0.011 ^b^
Tubular/papillary adenocarcinoma, NOS	150 (85.7)	73 (79.3)	77 (92.8)	
Poorly cohesive carcinoma, signet-ring cell type	25 (14.3)	19 (20.7)	6 (7.2)	
**Lauren’s classification**				0.024 ^b^
Intestinal	117 (66.9)	53 (57.6)	64 (77.1)	
Diffuse	40 (22.9)	27 (29.3)	13 (15.7)	
Indeterminate	18 (10.3)	12 (13.0)	6 (7.2)	
**Tumor grade**				0.556 ^b^
Well differentiated	29 (16.6)	13 (14.1)	16 (19.3)	
Moderately differentiated	79 (45.1)	41 (44.6)	38 (45.8)	
Poorly differentiated	67 (38.3)	38 (41.3)	29 (34.9)	
**Type of surgery**				0.762 ^b^
Subtotal gastrectomy	78 (44.6)	42 (45.7)	36 (43.4)	
Total gastrectomy	97 (55.4)	50 (54.3)	47 (56.6)	
**ypT**				0.881 ^b^
0	19 (10.9)	12 (13.0)	7 (8.4)	
1	29 (16.6)	16 (17.4)	13 (15.7)	
2	18 (10.3)	9 (9.8)	9 (10.8)	
3	65 (37.1)	33 (35.9)	32 (38.6)	
4a	44 (25.1)	22 (23.9)	22 (26.5)	
**ypN**				0.002 ^b^
0	75 (42.9)	47 (51.1)	28 (33.7)	
1	28 (16.0)	10 (10.9)	18 (21.7)	
2	47 (26.9)	17 (18.5)	30 (36.1)	
3	25 (14.3)	18 (19.6)	7 (8.4)	
**ypTNM stage**				0.419 ^b^
0	15 (8.6)	10 (10.9)	5 (6.0)	
1	35 (20.0)	21 (22.8)	14 (16.9)	
2	41 (23.4)	21 (22.8)	20 (24.1)	
3	84 (48.0)	40 (43.5)	44 (53.0)	
**Response to treatment**				0.429 ^b^
Complete response	15 (8.6)	10 (10.9)	5 (6.0)	
Near-complete response	33 (18.9)	20 (21.7)	13 (15.7)	
Partial response	45 (25.7)	22 (23.9)	23 (27.7)	
No response	82 (46.9)	40 (43.5)	42 (50.6)	
**Surgical margin**				0.480 ^c^
Negative (R0)	167 (95.4)	89 (96.7)	78 (94.0)	
Positive (R1)	8 (4.6)	3 (3.3)	5 (6.0)	
**Recurrence**				0.236 ^b^
No	103 (58.9)	58 (63.0)	45 (54.2)	
Yes	72 (41.1)	34 (37.0)	38 (45.8)	
**Mortality**				0.004 ^b^
Alive	100 (57.1)	62 (67.4)	38 (45.8)	
Dead	75 (42.9)	30 (32.6)	45 (54.2)	
**Anemia**				<0.001
Absent	86 (49.1)	79 (85.9)	7 (8.4)	
Present	89 (50.9)	13 (14.1)	76 (91.6)	

Data are presented as n (%) unless otherwise indicated. ^a^ Independent-samples *t*-test; ^b^ Pearson chi-square test; ^c^ Fisher’s exact test. *p* < 0.05 was considered statistically significant.

**Table 2:** Receiver operating characteristic (ROC) analysis of pretreatment hematological indices for the discrimination of overall mortality.

Index	AUC	95% CI	Cut-Off	Sensitivity (%)	Specificity (%)	*p*
HGB/RDW	0.608	0.524–0.693	≤0.85	60	62	0.014
LDH/ALB	0.588	0.503–0.674	≥4.63	54.7	54	0.046
PNI	0.586	0.499–0.673	≤48.20	57.3	57	0.049
PAR	0.58	0.494–0.665	≥7.05	56	55	0.041
RAR	0.579	0.494–0.664	≥0.37	54.7	59	0.045
ALR	0.569	0.483–0.655	≥21.80	54.7	54	0.12
MAR	0.552	0.466–0.639	≤0.14	57.3	51	0.235
SIRI	0.535	0.448–0.623	≤1.53	52	52	0.426
SII	0.534	0.446–0.622	≥748.80	54.7	55	0.447
NLR	0.53	0.433–0.618	≥2.75	56	56	0.491
NAR	0.529	0.442–0.615	≤1.26	53.3	54	0.519
PIV	0.517	0.429–0.606	≤425.65	50.7	50	0.694
CAR	0.513	0.425–0.600	≤1.23	52	52	0.773
LMR	0.513	0.426–0.600	≥3.05	52	53	0.771

ROC curve analysis; state variable: overall mortality. Cut-off values were determined by the maximum Youden index. AUC, area under the curve; CI, confidence interval; HGB/RDW, hemoglobin-to-red cell distribution width ratio; LDH/ALB, lactate dehydrogenase-to-albumin ratio; PNI, prognostic nutritional index; PAR, platelet-to-albumin ratio; RAR, RDW-to-albumin ratio; ALR, albumin-to-lymphocyte ratio; MAR, monocyte-to-albumin ratio; SIRI, systemic inflammation response index; SII, systemic immune–inflammation index; NLR, neutrophil-to-lymphocyte ratio; NAR, neutrophil-to-albumin ratio; PIV, pan-immune–inflammation value; CAR, C-reactive protein-to-albumin ratio; LMR, lymphocyte-to-monocyte ratio. *p* < 0.05 was considered statistically significant.

**Table 3:** Progression-free survival (PFS) according to clinical variables.

Variables	2-Year (%)	5-Year (%)	Median, Months (95% CI)	*p*
**Overall**	60.7	42.3	38.46 (16.13–60.79)	
**Age**				
<65	52.8	40.6	26.93 (5.81–48.04)	0.126
≥65	64.0	43.8	53.86 (23.02–84.70)	
**Sex**				
Male	53.2	41.3	26.93 (9.42–44.44)	0.140
Female	63.6	43.6	53.86 (26.95–80.78)	
**HGB/RDW**				
>0.85	69.4	53.7	62.20 (NR)	0.047
≤0.85	49.6	36.4	23.30 (9.73–36.86)	

Kaplan–Meier method, log-rank test. NR: not reached. *p* < 0.05 was considered statistically significant.

**Table 4:** Overall survival (OS) according to clinical variables.

Variables	2-Year (%)	5-Year (%)	Median, Months (95% CI)	*p*
**Overall**	68.4	39.6	43.50 (35.38–51.61)	
**Age**				
<65	70.7	44.8	43.50 (35.72–51.27)	0.841
≥65	67.7	38.3	45.16 (35.36–54.96)	
**Sex**				
Male	69.1	35.8	41.66 (34.56–58.35)	0.150
Female	56.9	28.6	38.08 (16.16–46.39)	
**HGB/RDW**				
>0.85	76.2	51.6	NR	0.003
≤0.85	59.6	27.7	35.06 (23.16–46.96)	

Kaplan–Meier method, log-rank test. NR: not reached. *p* < 0.05 was considered statistically significant.

**Table 5:** Multivariate Cox regression analysis for overall survival.

Variables	HR (95% CI)	*p*
**Sex**		
Male	Reference	
Female	1.48 (0.89–2.48)	0.131
**HGB/RDW**		
>0.85	Reference	
≤0.85	1.88 (1.16–3.06)	0.011
**ypN category**	1.63 (1.30–2.03)	<0.001
**Histological type**		
Tubular/papillary NOS	Reference	
Poorly cohesive, signet-ring	0.86 (0.40–1.82)	0.685
**Lauren’s classification**		
Intestinal	Reference	
Diffuse	1.83 (1.04–3.22)	0.036
Indeterminate	0.88 (0.30–2.57)	0.819

HR: hazard ratio; CI: confidence interval. *p* < 0.05 was considered statistically significant.

**Table 6:** Multivariate Cox regression analysis for progression-free survival.

Variables	HR (95% CI)	*p*
**Age**		
<65	Reference	
≥65	0.94 (0.62–1.41)	0.757
**Sex**		
Male	Reference	
Female	1.32 (0.85–2.03)	0.216
**HGB/RDW**		
>0.85	Reference	
≤0.85	1.79 (1.18–2.72)	0.006
**ypN category**	1.77 (1.46–2.14)	<0.001
**Histological type**		
Tubular/papillary NOS	Reference	
Poorly cohesive, signet-ring	1.15 (0.58–2.27)	0.684
**Lauren’s classification**		
Intestinal	Reference	
Diffuse	1.09 (0.64–1.87)	0.751
Indeterminate	1.18 (0.54–2.57)	0.683

HR: hazard ratio; CI: confidence interval. *p* < 0.05 was considered statistically significant.

## Data Availability

The data that support the findings of this study are available from the corresponding author upon reasonable request. The data are not publicly available due to institutional data protection regulations.

## Abbreviations

The following abbreviations are used in this manuscript:

FLOT	fluorouracil, leucovorin, oxaliplatin, and docetaxel
OS	overall survival
PFS	progression-free survival
HR	hazard ratio
CI	confidence interval
HGB/RDW	hemoglobin-to-red cell distribution width ratio
yp	post-neoadjuvant
LDH/ALB	lactate dehydrogenase-to-albumin ratio
PNI	prognostic nutritional index
PAR	platelet-to-albumin ratio
RAR	RDW-to-albumin ratio
ALR	albumin-to-lymphocyte ratio
MAR	monocyte-to-albumin ratio
SIRI	systemic inflammation response index
SII	systemic immune–inflammation index
NLR	neutrophil-to-lymphocyte ratio
NAR	neutrophil-to-albumin ratio
PIV	pan-immune–inflammation value
CAR	C-reactive protein-to-albumin ratio
LMR	lymphocyte-to-monocyte ratio
